# Supplementary figures and images for: A sticky Poisson Hidden Markov Model for solving the problem of over-segmentation and rapid state switching in cortical datasets
Source: PLoS One. 2025 Jul 1;20(7):e0325979. doi: 10.1371/journal.pone.0325979 (PMC12212568; doi:10.1371/journal.pone.0325979)

A

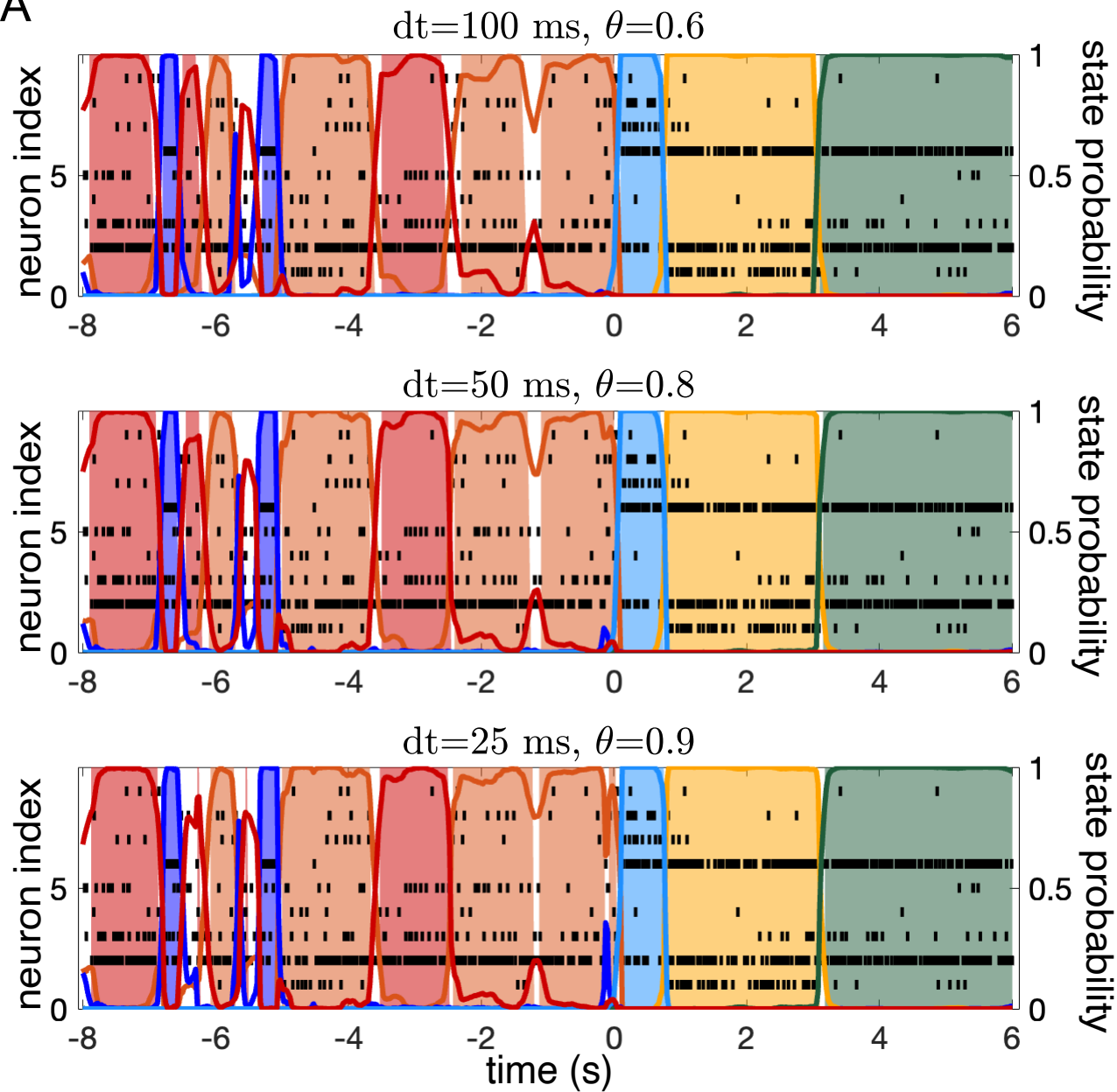

B

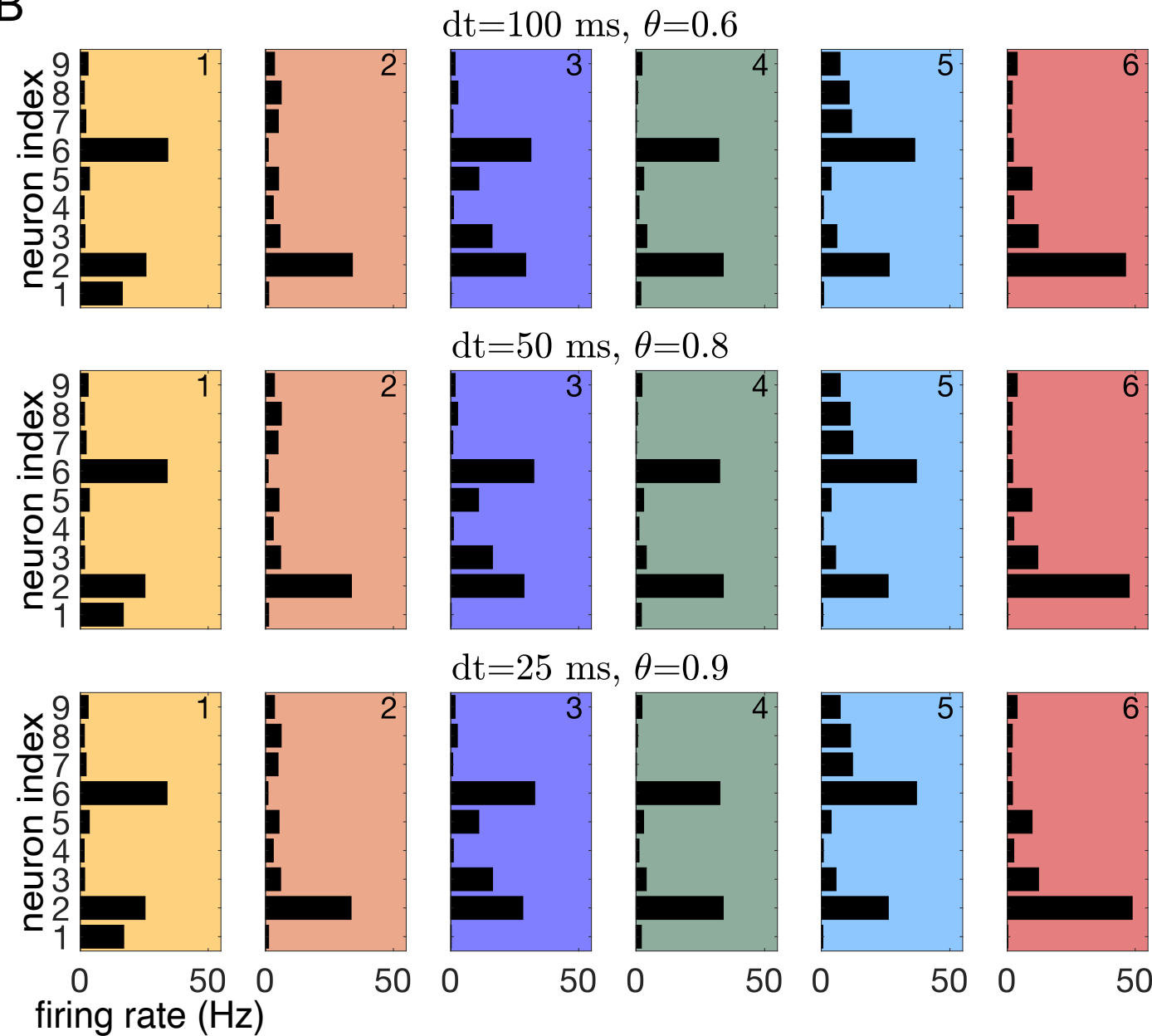

Supplement: S1 Fig — A: sPHMM decoding of an EXP dataset with 9 neurons after training with three different sets of (dt, θ) values which correspond to a mean state duration of 250 ms. The three sets of parameters were, from top to bottom: (dt=100 ms,θ=0.6); (dt=50 ms,θ=0.8);(dt=25 ms,θ=0.9). B: The hidden states corresponding to the results shown in panel A (same color code). Each panel shows the inferred firing rates for each state for the three different sets of parameters. No appreciable difference is visible across states. The average Euclidean distance among corresponding states is 0.9 spikes/s and is less than 2.6 spikes/s in all cases. (PDF) [file pone.0325979.s001.pdf]

**A**

100 initial conditions

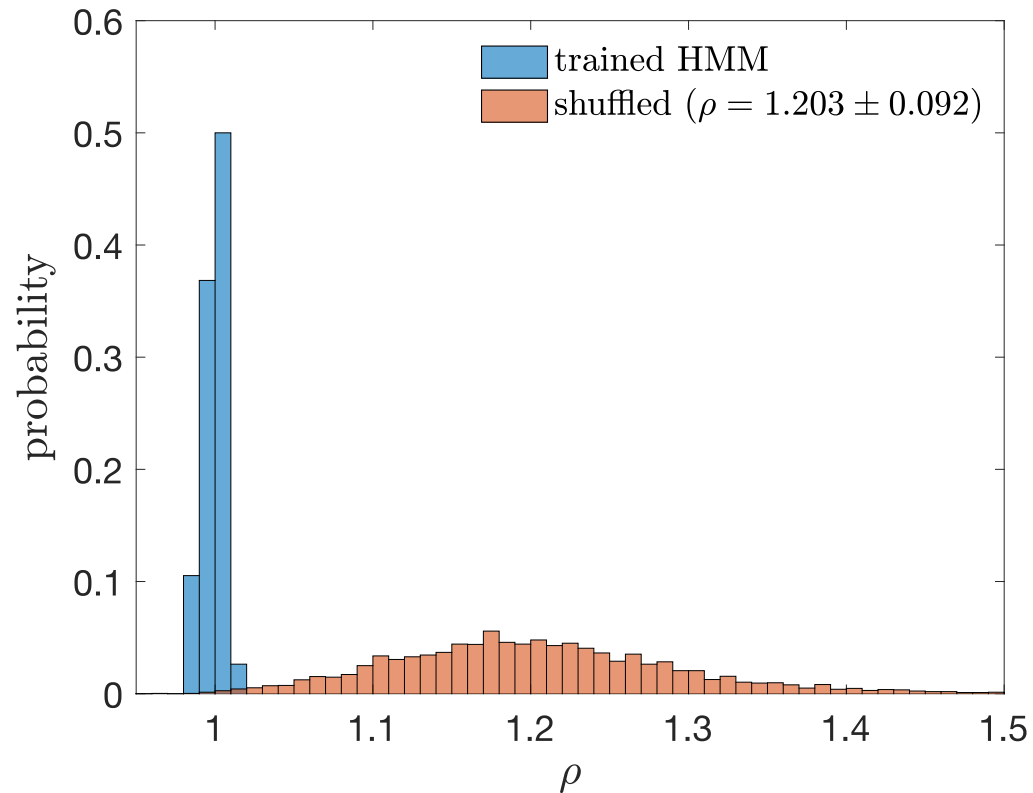**B**

1000 initial conditions

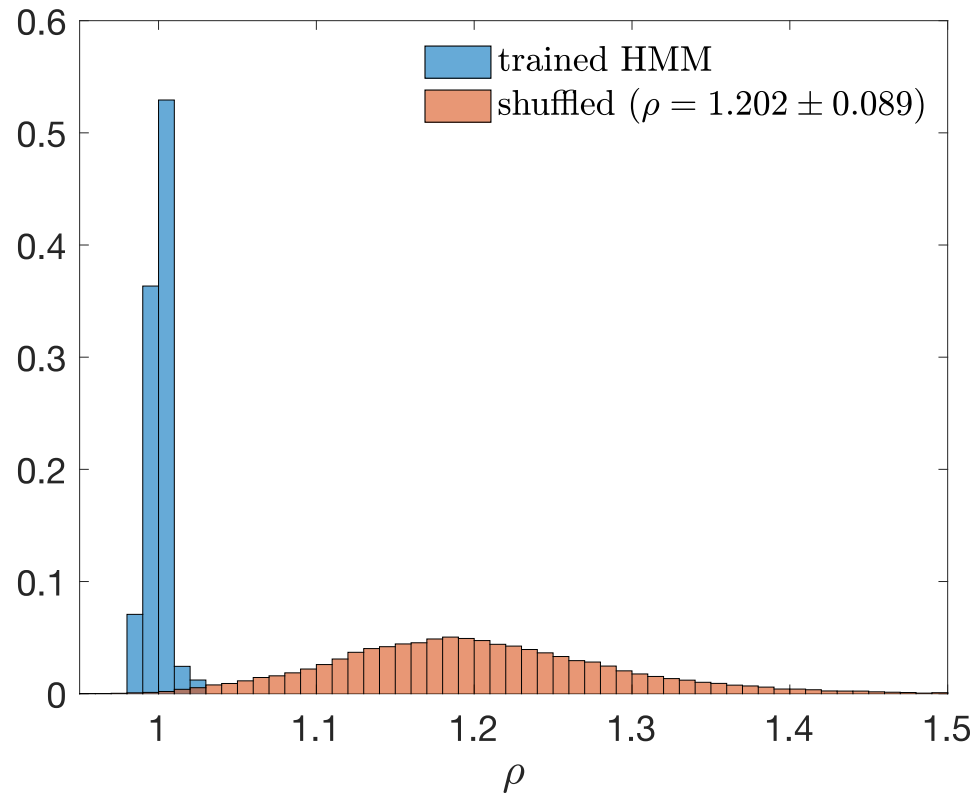

Supplement: S2 Fig — ρ=D(Θ)/D(Θ*) for the models in Fig 11 of the main manuscript, where Θ* is the best model trained with 1,000 initial conditions (in lieu of the true model). A: Histogram of the ρ values when Θ is the model trained with 100 initial conditions (blue) compared with the histogram of the same model after shuffling its states’ firing rates and off-diagonal transition probabilities (100 shuffles for each trained model). The ρ values were narrowly distributed around 1, with a probability of 0.0082 of getting the largest ρ value (or a smaller value) under the shuffled model. B: Same as panel A with Θ being the model trained with 1,000 initial conditions. The probability of getting the trained ρ values under the shuffled model is 0.0098. (PDF) [file pone.0325979.s002.pdf]
