# Supplementary material for: A sticky Poisson Hidden Markov Model for solving the problem of over-segmentation and rapid state switching in cortical datasets
Source: PLoS One. 2025 Jul 1;20(7):e0325979. doi: 10.1371/journal.pone.0325979 (PMC12212568; doi:10.1371/journal.pone.0325979)
Supplement: S1 Appendix — (PDF) [file pone.0325979.s003.pdf]

S1 Appendix. State persistence and self-transition probabilities

In this Appendix we discuss the link between the self-transition probabilities γ_{ii} and the temporal persistence of the hidden states. Let S_t be the state in bin t and define the holding time H_i as the number of bins during which the state remains i, i.e., $\{H_i(t) = n\}$ is the event $\{S_{t+1} = i, S_{t+2} = i, ..., S_{t+n-1} = i, S_{t+n} \neq i\}$. Using the Markov property and the definition $\gamma_{ii}(t) = P(S_{t+1} = i|S_t = i)$,

$$P(H_i(t) = n|S_t = i) = P(S_{t+n} \neq i, S_{t+n-1} = i, ..., S_{t+2} = i, S_{t+1} = i|S_t = i) = (1 - \gamma_{ii})\gamma_{ii}^{n-1}, \quad (1)$$

and does not depend on the initial time bin t because the chain is assumed homogeneous ($\gamma_{ii}(t)$ does not depend on t). Hence we can write simply H_i. Applying this formula, we learn that the self-transition probability γ_{ii} is also the probability that a state persists for 2 or more time bins:

$$P(H_i \geq 2|S_t = i) = 1 - P(H_i = 1|S_t = i) = \gamma_{ii}. \quad (2)$$

For example, for a 50 ms bin and $\gamma_{ii} \geq 0.8$ (as enforced by the sHMM), there is a probability ≥ 0.8 that a state persists for 100 ms or longer.

We can also estimate the probability of remaining in the same state for n or more time bins, with $n > 2$. $P(H_i \geq n|S_t = i)$ equals the probability of remaining in i for at least $n - 1$ steps, times the probability of making a self-transition, i.e.,

$$P(H_i \geq n|S_t = i) = \gamma_{ii} P(H_i \geq n - 1|S_t = i). \quad (3)$$

Iterating this equality and using $P(H_i \geq 2|S_t = i) = \gamma_{ii}$ we get

$$P(H_i \geq n|S_t = i) = \gamma_{ii}^{n-1} \quad \text{for all } n \geq 2. \quad (4)$$

For example, for 20 ms bins, the probability of state durations $\geq$ 100 ms is $\gamma_{ii}^4 \geq 0.8^4 = 0.4096$. If $\gamma_{ii} \geq 0.9$, this value would be 0.6561.

We finally consider the average time spent in state i. From Eq. 1 this is

$$\langle H_i|S_t = i\rangle = \sum_{n=1}^{\infty} nP(H_i = n|S_t = i) = \sum_{n=1}^{\infty} n(1 - \gamma_{ii})\gamma_{ii}^{n-1} = \frac{1}{1 - \gamma_{ii}}, \quad (5)$$

where we have used $\sum_{n=1}^{\infty} nx^{n-1} = \frac{1}{(1-x)^2}$ for $|x| < 1$, and $\langle\cdot\rangle$ denotes expectation.

From Eq. 5 we see that if $\gamma_{ii} \geq 0.8$, the average time spent in state i is at least 5 time bins, which ranges from 100 ms to 500 ms for bins of 20 to 100 ms.

Inverting Eq. 5 gives the target γ_{ii} value to be used in the sPHMM and DPHMM algorithms, based on the desired mean state durations. Specifically, if $\langle T\rangle_{min}$ is the minimal desired mean state duration and dt is the bin width, we must impose

$$\gamma_{ii} \geq \theta = 1 - \frac{dt}{\langle T\rangle_{min}}. \quad (6)$$
