# Supplementary material for: A sticky Poisson Hidden Markov Model for solving the problem of over-segmentation and rapid state switching in cortical datasets
Source: PLoS One. 2025 Jul 1;20(7):e0325979. doi: 10.1371/journal.pone.0325979 (PMC12212568; doi:10.1371/journal.pone.0325979)
Supplement: S2 Appendix — (PDF) [file pone.0325979.s004.pdf]

S2 Appendix. The expectation-maximization algorithm for HMMs

In this appendix we describe how the EM algorithm is applied to an HMM to obtain estimates of its parameters. Given the HMM model with parameters $\Theta = \{\pi, \Gamma, E\}$, the goal is to maximize the likelihood $P(O_{1:T}|\Theta)$, or equivalently its logarithm:

$$\ln P(O_{1:T}|\Theta) = \ln \sum_{\{S\}} P(O_{1:T}, S_{1:T}|\Theta), \tag{1}$$

where $\{S\}$ represents all possible sequences of hidden states $S_{1:T}$.

In the following, to lighten the notation, we write S for $S_{1:T}$ and O for $O_{1:T}$.

Even taking into account the Markov property, computing naïvely the LL in Eq. 1 requires an exponential number of calculations ($\propto TM^T$; see e.g. Rabiner, 1989). The EM algorithm for HMMs is a tractable, iterative scheme that allows to re-estimate the parameters of the model at each step such that the likelihood $P(O|\Theta)$ does not decrease. The resulting re-estimation procedure is known as the Baum-Welch algorithm (Baum et al., 1970; Welch, 2003).

To obtain the algorithm, one starts from the auxiliary function

$$Q(\Theta, \hat{\Theta}) = \sum_S P(S|O, \Theta) \ln P(S, O|\hat{\Theta}) \doteq \langle \ln P(S, O|\hat{\Theta}) \rangle_{S \sim P(S|O,\Theta)}, \tag{2}$$

which is the expectation of $\ln P(S, O|\hat{\Theta})$, the log-probability of the complete data given a parameter vector $\hat{\Theta}$, with respect to $P(S|O, \Theta)$, the probability of the hidden states given the observations O and the current estimate of the parameters, Θ. It is a classical result that, under some rather general conditions (Baum and Eagon, 1967; Baum et al., 1970; Baum, 1972), maximizing $Q(\Theta, \hat{\Theta})$ over $\hat{\Theta}$ guarantees that

$$P(O|\hat{\Theta}) \geq P(O|\Theta). \tag{3}$$

Moreover, if the Q function is strictly maximized, so is the likelihood. If $P(O|\hat{\Theta})$ converges, the bound above insures that it will converge to a local maximum (or a saddle) of the likelihood function (see also Gupta and Chen, 2010). To maximize $Q(\Theta, \hat{\Theta})$, we first write down its current estimate, i.e., the average of $\ln P(S, O|\hat{\Theta})$ with respect to $P(S|O, \Theta)$, and then we maximize the result over $\hat{\Theta}$. These are called the E-step and the M-step, respectively, and are described below.

E-step

In the E-step we build the function $Q(\Theta, \hat{\Theta})$. Exploiting the Markovian nature of the process, $\ln P(S, O|\hat{\Theta})$ is given by

$$\ln P(S, O|\hat{\Theta}) = \ln \left[\hat{\pi}_{S_1} \hat{e}_{S_1}(O_1) \hat{\gamma}_{S_1 S_2} \hat{e}_{S_2}(O_2) \ \dots \ \hat{\gamma}_{S_{T-1} S_T} \hat{e}_{S_T}(O_T) \right] \tag{4}$$

$$= \ln \left[\hat{\pi}_{S_1} \prod_{t=1}^{T-1} \hat{\gamma}_{S_t, S_{t+1}} \prod_{t=1}^{T} \hat{e}_{S_t}(O_t) \right] \tag{5}$$

$$= \ln \hat{\pi}_{S_1} + \sum_{t=1}^{T-1} \ln \hat{\gamma}_{S_t, S_{t+1}} + \sum_{t=1}^{T} \ln \hat{e}_{S_t}(O_t). \tag{6}$$

The function $Q(\Theta, \hat{\Theta})$ is the expectation of the above expression with respect to $P(S|O,\Theta)$, that is,

$$Q(\Theta, \hat{\Theta}) = \langle \ln \hat{\pi}_{S_1} \rangle_{S|O,\Theta} + \langle \sum_{t=1}^{T-1} \ln \hat{\gamma}_{S_t, S_{t+1}} \rangle_{S|O,\Theta} + \langle \sum_{t=1}^{T} \ln \hat{e}_{S_t}(O_t) \rangle_{S|O,\Theta} \tag{7}$$

$$= \sum_{i=1}^{m} q_i(1) \ln \hat{\pi}_i + \sum_{i,j=1}^{m} \sum_{t=1}^{T-1} \xi_{ij}(t) \ln \hat{\gamma}_{ij} + \sum_{i=1}^{m} \sum_{t=1}^{T} q_i(t) \ln \hat{e}_i(O_t), \tag{8}$$

where the symbols q_i and ξ_{ij} are defined as

$$q_i(t) = P(S_t = i|O, \Theta), \tag{9}$$

$$\xi_{ij}(t) = P(S_t = i, S_{t+1} = j|O, \Theta), \tag{10}$$

and note that $q_i(t) = \sum_{j=1}^{m} \xi_{ij}(t)$.

Building the function $Q(\Theta, \hat{\Theta})$, or equivalently the posterior probability $P(S|O,\Theta)$, represents the E-step of the EM algorithm. In this case, the E-step boils down to the computation of q_i and ξ_{ij}. This is done efficiently with the forward-backward algorithm described below.

M-step

Given the current estimates for q_i and ξ_{ij}, in the M-step we maximize Eq. 8 with respect to $\hat{\pi}_i$, $\hat{\gamma}_{ij}$ and $\hat{e}_i(O_t)$. Note that these are constrained optimizations because $\sum_i \hat{\pi}_i = 1$, $\sum_j \hat{\gamma}_{ij} = 1$ and $\sum_k \hat{e}_i(k) = 1$, and therefore Lagrange multipliers are required. From the first two optimizations we obtain

$$\hat{\pi}_i = q_i(1), \quad i = 1, ..., , m \tag{11}$$

$$\hat{\gamma}_{ij} = \frac{\sum_{t=1}^{T-1} \xi_{ij}(t)}{\sum_{t=1}^{T-1} q_i(t)} = \frac{\text{expected number of transitions } i \to j}{\text{expected number of transitions from state } i}. \tag{12}$$

The optimization with respect to the emission probabilities, $\hat{e}_i$, depends on the observation model. For the MHMM this gives

$$\hat{e}_i(n) = \frac{\sum_{t=1}^{T} q_i(t)\delta_n(t)}{\sum_{t=1}^{T} q_i(t)} = \frac{\text{expected number of observations of symbol } n \text{ while in state } i}{\text{expected number of occurrences of state } i}, \tag{13}$$

where n indexes the observation symbols and $\delta_n(t) = 1$ if symbol n is observed at time t, and zero otherwise.

For the Poisson-HMMs, the emission probabilities depend on the firing rates λ_{ni}; maximization with respect to $\hat{\lambda}_{ni}$ gives

$$\hat{\lambda}_{ni} \Delta t = \frac{\sum_{t=1}^{T} q_i(t) k_n(t)}{\sum_{t=1}^{T} q_i(t)}, \tag{14}$$

in which $k_n(t)$ is the spike count of neuron n in bin t. The re-estimated emission probabilities are therefore

$$\hat{e}_i(\boldsymbol{k}(t)) = \prod_{n=1}^{N} \frac{(\hat{\lambda}_{ni} \Delta t)^{k_n(t)} \, e^{-\hat{\lambda}_{ni} \Delta t}}{k_n(t)!}. \tag{15}$$

In the case of multiple independent trials, the log-likelihood Eq. 6 contains a sum over all bins across all trials; maximization then requires adding the numerators and denominators across trials; for example, for $\mathcal{T}$ trials, Eq. 12 becomes

$$\hat{\gamma}_{ij} = \frac{\sum_{r=1}^{\mathcal{T}} \sum_{t=1}^{T-1} \xi_{ij}^{r}(t)}{\sum_{r=1}^{\mathcal{T}} \sum_{t=1}^{T-1} q_{i}^{r}(t)} = \frac{\text{expected number of transitions } i \to j \text{ across all trials}}{\text{expected number of transitions from state } i \text{ across all trials}}. \tag{16}$$

The forward-backward algorithm

Both the likelihood of the model, $P(O|\Theta)$, and the quantities $q_i(t)$ and $\xi_{ij}(t)$ require an exponential number of computations, and therefore an efficient method to compute these quantities is necessary. This can be done via the forward and backward probabilities, defined as

$$\alpha_i(t) = P(O_{1:t}, S_t = i), \tag{17}$$

$$\beta_i(t) = P(O_{t+1:T}|S_t = i). \tag{18}$$

Due to the Markov property, these probabilities can be computed recursively from

$$\alpha_i(1) = \pi_i e_i(O_1), \quad 1 \le i \le m, \tag{19}$$

$$\alpha_j(t+1) = \sum_{i=1}^{m} \alpha_i(t)\gamma_{ij}\, e_j(O_{t+1}), \quad 1 \le t \le T-1, \quad 1 \le j \le m, \tag{20}$$

and

$$\beta_i(T) \doteq 1, \quad 1 \le i \le m, \tag{21}$$

$$\beta_i(t) = \sum_{j=1}^{m} \gamma_{ij} e_j(O_{t+1})\beta_j(t+1), \quad t = T-1, T-2, ..., 1, \quad 1 \le j \le m. \tag{22}$$

These computations are tractable because they require only $\propto M^2 T$ calculations compared to the $\propto TM^T$ calculations involved in a naïve computation of $P(O|\Theta)$ (Rabiner, 1989). In terms of α and β, we have

$$P(O|\Theta) = \sum_{i=1}^{m} \alpha_i(t)\beta_i(t) = \sum_{i=1}^{m} \alpha_i(T), \tag{23}$$

$$q_i(t) = \frac{P(S_t = i, O|\Theta)}{P(O|\Theta)} = \frac{\alpha_i(t)\beta_i(t)}{P(O|\Theta)}, \tag{24}$$

$$\xi_{ij}(t) = \frac{P(S_t = i, S_{t+1} = j, O|\Theta)}{P(O|\Theta)} = \frac{\alpha_i(t)\gamma_{ij} e_j(O_{t+1})\beta_j(t+1)}{P(O|\Theta)}. \tag{25}$$

We see that the forward-backward algorithm is the 'soul' of EM for HMMs.

The EM algorithm with Dirichlet prior

In the presence of a prior, instead of the likelihood we seek to maximize the posterior probability

$$P(\hat{\Theta}|O) \propto P(O|\hat{\Theta})P(\hat{\Theta}), \tag{26}$$

which is equivalent to maximizing

$$\ln P(O|\hat{\Theta}) + \ln P(\hat{\Theta}). \tag{27}$$

In the E step, we again replace $\ln P(O|\hat{\Theta})$ with $Q(\Theta, \hat{\Theta})$ (see Eq. 2); in the M step we maximize

$$Q(\Theta, \hat{\Theta}) + \ln P(\hat{\Theta}) \tag{28}$$

with respect to $\hat{\Theta}$. It can be proved that, after each expectation-maximization step, $P(\hat{\Theta}|O) \geq P(\Theta|O)$ (see e.g. Gupta and Chen, 2010), hence each iteration of the algorithm tends to increase (or at least, not decrease) the posterior probability.

In the DPHMM, $P(\hat{\Theta})$ contains only our Dirichlet prior on the transition probabilities, $\prod_i^m P(\hat{\Gamma}_i|\boldsymbol{a}_i) \propto \prod_{ij}^m \gamma_{ij}^{a_{ij}-1}$ (see the main text); therefore, up to additive terms that do not depend on $\hat{\gamma}_{ij}$ (or other model parameters),

$$\ln P(\hat{\Theta}) = \sum_{i=1}^{m}\sum_{j=1}^{m}(a_{ij}-1)\ln\hat{\gamma}_{ij}. \tag{29}$$

$Q(\Theta, \hat{\Theta})$ is given by Eq. 8 as before.

The E-step amounts again to the estimation of q_i and ξ_{ij}, which is achieved with the same forward-backward algorithm presented in Sec. . The M-step differs only in the re-estimation of γ_{ij}, because the prior depends only on $\hat{\gamma}_{ij}$. Thus, the only difference with Sec. is in the function to be maximized with respect to $\hat{\gamma}_{ij}$, which now reads

$$\sum_{i,j=1}^{m}\sum_{t=1}^{T-1}\xi_{ij}(t)\ln\hat{\gamma}_{ij} + \sum_{i,j=1}^{m}(a_{ij}-1)\ln\hat{\gamma}_{ij} + \sum_{i=1}^{m}\eta_i(\sum_{j=1}^{m}\hat{\gamma}_{ij}-1), \tag{30}$$

where η_i are Lagrange multipliers. Performing the maximization one obtains the re-estimation formula of the main text:

$$\hat{\gamma}_{ij} = \frac{\sum_{t=1}^{T-1}\xi_{ij}(t) + a_{ij} - 1}{\sum_{t=1}^{T-1}q_i(t) + \sum_{j=1}^{m}(a_{ij}-1)}. \tag{31}$$

In the presence of $\mathcal{T}$ independent trials, a sum over trials must be added in the first term of Eq. 30, giving

$$\hat{\gamma}_{ij} = \frac{\sum_{r=1}^{\mathcal{T}}\sum_{t=1}^{T-1}\xi_{ij}^r(t) + a_{ij} - 1}{\sum_{r=1}^{\mathcal{T}}\sum_{t=1}^{T-1}q_i^r(t) + \sum_{j=1}^{m}(a_{ij}-1)}. \tag{32}$$

The log-posterior, $\ln P(\hat{\Theta}|O)$, can be efficiently computed (up to an irrelevant additive constant) using Eqs. 23 and 29.

References

Baum L (1972) An inequality and associated maximization technique in statistical estimation for probabilistic functions of Markov processes. *Inequalities* 3: 1–8.

Baum LE, Eagon J (1967) An inequality with applications to statistical estimation for probabilistic functions of Markov processes and to a model for ecology. *Bull. Amer. Math. Soc.* 73: 360–363.

Baum LE, Petrie T, Soules G, Weiss N (1970) A maximization technique occurring in the statistical analysis of probabilistic functions of Markov chains. *The Annals of Mathematical Statistics* 41: 164–171.

Gupta MR, Chen Y (2010) Theory and use of the EM algorithm. *Foundations and Trends® in Signal Processing* 4: 223–296.

Rabiner L (1989) A tutorial on hidden Markov models and selected applications in speech recognition. *Proceedings of the IEEE* 77: 257–286.

Welch LR (2003) Hidden Markov Models and the Baum-Welch Algorithm. *IEEE Information Theory Society Newsletter* 53: 1–13.
