# Supplementary material for: A sticky Poisson Hidden Markov Model for solving the problem of over-segmentation and rapid state switching in cortical datasets
Source: PLoS One. 2025 Jul 1;20(7):e0325979. doi: 10.1371/journal.pone.0325979 (PMC12212568; doi:10.1371/journal.pone.0325979)
Supplement: Attachment — Submitted filename: HMM_reply_letter.pdf [file pone.0325979.s005.pdf]

Reply letter

Dear Editor,

we wish to thank the reviewers for their useful comments and feedback. We believe that we have addressed all queries and improved our paper, which is stronger as a result. This effort resulted in quite a bit of additional work. In particular, we have introduced a new method to compare two HMMs (for example, to see if they converge to the same model when using different numbers of initial conditions). This method can be used to answer many questions, as we show below in our point-to-point replies, and we wished we had it when previously working on HMM analyses. We thank the reviewers for inspiring us to produce such a method.

To allow for the most important points of our new analyses to be available with the published manuscript, we have now added two new sections to the manuscript (Sec. 2.7 of Results, "Full model comparison" and Sec. 4.6 of Methods, "Model comparison") and 2 supplementary figures. We report in the point-to-point replies the details of what has been included in the main manuscript and the supplementary material. Any changes in the main manuscript have been highlighted in red ink.

Reviewer 1

The paper is in most ways thorough and clear, with good data backing up the proposed methods for producing optimal HMMs of data. There are a few things I would like to see mentioned minimally, and ideally carried out as time permits:

We thank the reviewer for the positive evaluation of our paper.

1) While the authors mention a threshold other than 0.8 could be used, I think it important to connect the threshold to the time-bin being used for the model. For example, with 5ms bins a minimum of 0.8 for self-transitions means a maximum of a 1/5 probability per 5ms of a transition. If time bins were 10ms the minimum would be about 0.64 and with 1ms time bins, on the order of 0.96 (approx. 1/25 probability per time bin). This should be discussed and ideally a demonstration that the method with altered time bin and correspondingly altered threshold leads to the same results, whereas a very different time bin but unaltered threshold would be less ideal.

The connection between threshold and time bin is discussed in Appendix A, where we give the mean state duration and other quantities as a function of the threshold. We have now added the explicit relationship between the threshold, the time bin, and the minimal desired mean state duration, which is $\theta = 1 - dt/\langle T \rangle$. The sticky algorithm insures that $\gamma_{ii} > \theta$, hence the mean state duration is equal or larger than $\langle T \rangle$.

To answer this query, we have run HMM training with different parameters at parity of $\langle T \rangle$. In general, the sPHMM algorithm is robust to changes in dt and θ within a reasonable range that is required for any HMM to work – i.e., dt not too large (otherwise some transitions may be lost) and θ not too small (otherwise rapid state switching may result). We show an example in Fig. 1A below where we set $\langle T \rangle = 250$ms and train the model with three sets of parameters. The figure shows no appreciable difference after decoding. In Fig. 1B we show that the inferred hidden states are indeed the same.

In the revised manuscript, we report these results in section 2.4 and include Fig. 1 as Supplementary Figure 1.

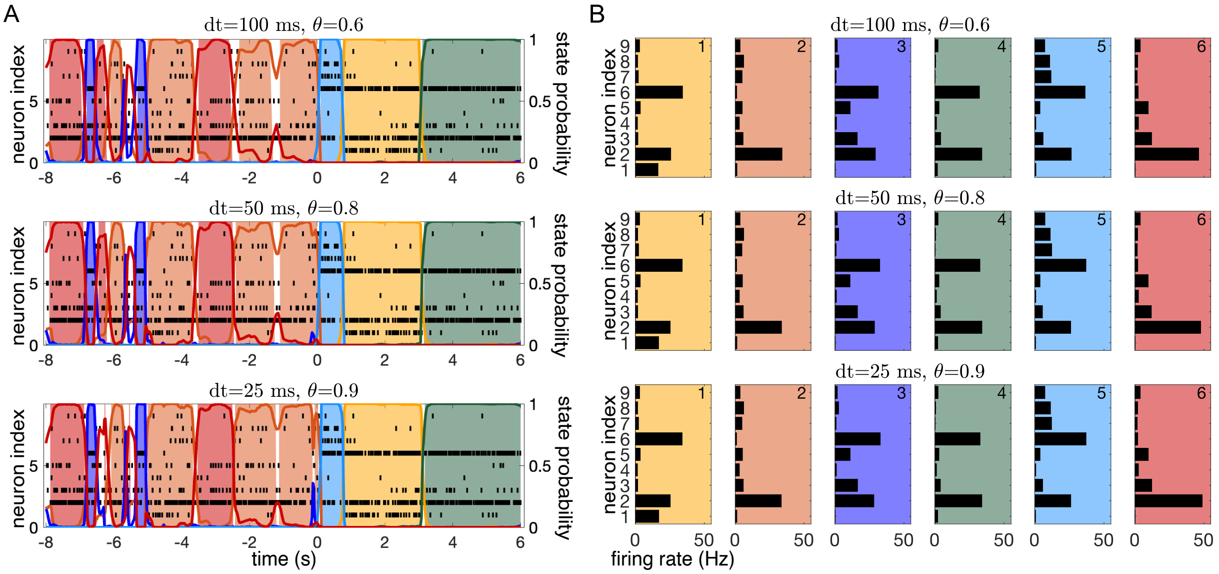

Figure 1: A) sPHMM decoding of an EXP dataset with 9 neurons after training with three different sets of (dt, θ) values which correspond to a mean state duration of 250 ms. The three sets of parameters were, from top to bottom: ($dt = 100$ ms, $\theta = 0.6$); ($dt = 50$ ms, $\theta = 0.8$); ($dt = 25$ ms, $\theta = 0.9$). B) The hidden states corresponding to the results shown in panel A (same color code). Each panel shows the inferred firing rates for each state for the three different sets of parameters. No appreciable difference is visible across states. The average Euclidean distance among corresponding states is 0.9 spikes/s and is less than 2.6 spikes/s in all cases.

2) Throughout the authors use the measure of number of states extracted as a measure of the validity of the model. Whereas the model produces firing rates as a function of time on each trial. It would be nice to show that the "correct" no. of states better matches these rates vs time when known – or how well transition times are captured.

To answer this query we need a way to quantify the agreement on state identity, state transition and state transition times between two models. To provide such quantification, we introduced a new metric ρ that measures the 'distance' between two models for a given dataset,

$$\rho(\Theta_1, \Theta_2) = \frac{D(\Theta_1)}{D(\Theta_2)}, \tag{1}$$

where $\Theta_i = (\Lambda_i, \Gamma_i)$ are the parameters of trained HMM models that need comparison. $D(\Theta)$ is defined

as the sum of squared differences between the observed and inferred spike counts in each bin:

$$D(\Theta) = \sum_{k=1}^{K} \sum_{t=1}^{T_k/dt} \sum_{n=1}^{N} (X_k(n,t) - f_{\Theta,k}(n,t)\, dt)^2, \tag{2}$$

where $X_k(n,t)$ is the spike count of neuron n in time bin t in trial k (lasting T_k) while $f_{\Theta,k}(n,t)$ is the model-inferred firing rate of the same neuron in the same bin, obtained from the decoded state in that bin. Each bin was decoded using posterior decoding, assigning the bin to the state with the largest posterior probability in that bin. $D(\Theta)$ is computed on a validation set not used for training.

$D(\Theta)$ measures the irreducible variability of the data when the latter has been generated by an HMM with parameters Θ. If does so by combining information about both the firing rate vectors defining the hidden states and the state transitions. If two models have the same states, the same state transitions, and the same transition times on a given dataset, we expect $\rho \approx 1$. When a true model is available, we set $\Theta_2 = \Theta_{true}$ and expect $\rho \geq 1$. In that case, $D(\Theta_{true})$ is computed after decoding the data with the model used to generate it (to account for the variability due to state transitions occurring inside a bin).

We show in Fig. 2A the ratio $\rho(m) = \rho(\Theta(m), \Theta_{true})$ for an sPHMM trained on an MMPP dataset with 20 neurons and 10 states, for which the true model is available. The trained model was trained with $m = 2, \ldots, 12$ states (the model did not converge for $m > 12$). $\rho(m)$ decreases for $m \leq 10$ and reaches $\rho \approx 1$ for $m \geq 10$. $\rho(m) > 1$ for $m < 10$, indicating that the model $\Theta(m)$ did not capture the state transition times and/or the firing rates generated by the true model as well as the model with $m = 10$. For comparison, we show in the same figure the ρ value for $m = 10$ obtained after randomly shuffling the states' firing rates and the off-diagonal transition probabilities. This is the value expected by chance when comparing the true model with a model with the same firing rates and transition rates as the test model with $m = 10$.

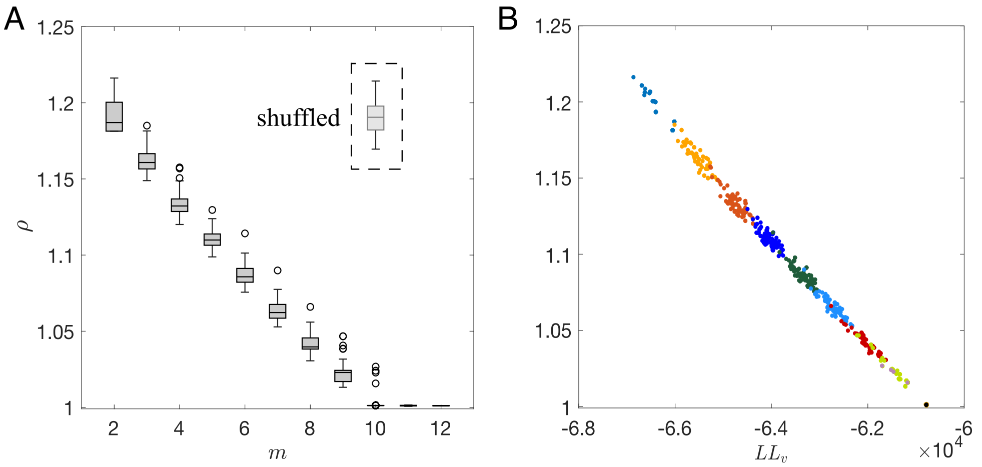

Figure 2: A) $\rho(m) = \rho(\Theta(m), \Theta_{true})$ for an sPHMM with m states trained on an MMPP dataset with 20 neurons and 10 states (50 trials, split into 40 and 10 in the training and validation sets, respectively). ρ decreases with m until m reaches the true number of states $m^* = 10$. For each m, 100 HMMs were trained with different random initial guesses for the model parameters. No HMM model converged when $m > 12$. *Dashed box:* the ρ value for $m = 10$ after randomly shuffling the states' firing rates and the off-diagonal transition probabilities. B) Log-likelihood on validation sets increases as a function of m and is highly anti-correlated to $\rho(m)$.

The values obtained for ρ vary across a short range (from 1 to ≈ 1.2), suggesting that ρ values slightly larger than 1 already indicate a meaningful mismatch between the models. To get a feeling for the meaning of the numerical ρ values, we show in Fig. 3 the decoding comparison between the true model and the models with $m = 5, 10$ and 12. The model with $m = 5$ gets most state transitions right, but it doesn't have enough states, therefore it mislabels some of the states. It gives $\rho \approx 1.1$. The model with $m = 10$ practically gives identical results to the true model and it gives $\rho = 1$. The overfit model with $m = 12$ is similarly good and also gives $\rho = 1$, but it is occasionally undecided on how to classify some bins (white space prior to grey state). This phenomenon occurs due to the 'duplication' of states in an overfit model, as we show next (henceforth, we refer exclusively to models that do no produce rapid state switching, otherwise some of the arguments presented below may not apply).

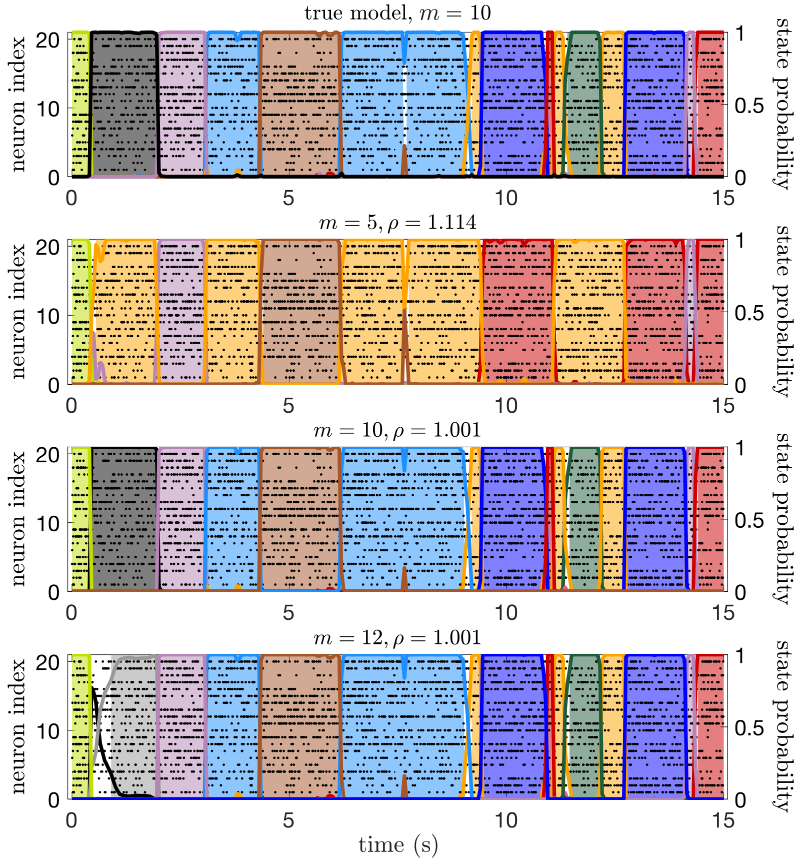

Figure 3: sPHMM's posterior decoding of the best HMM models (i.e., having the largest likelihood on the training set) trained with 5, 10, and 12 states compared with the true model (having 10 states) on an MMPP dataset with 20 neurons and 10 states (same as Fig. 2).

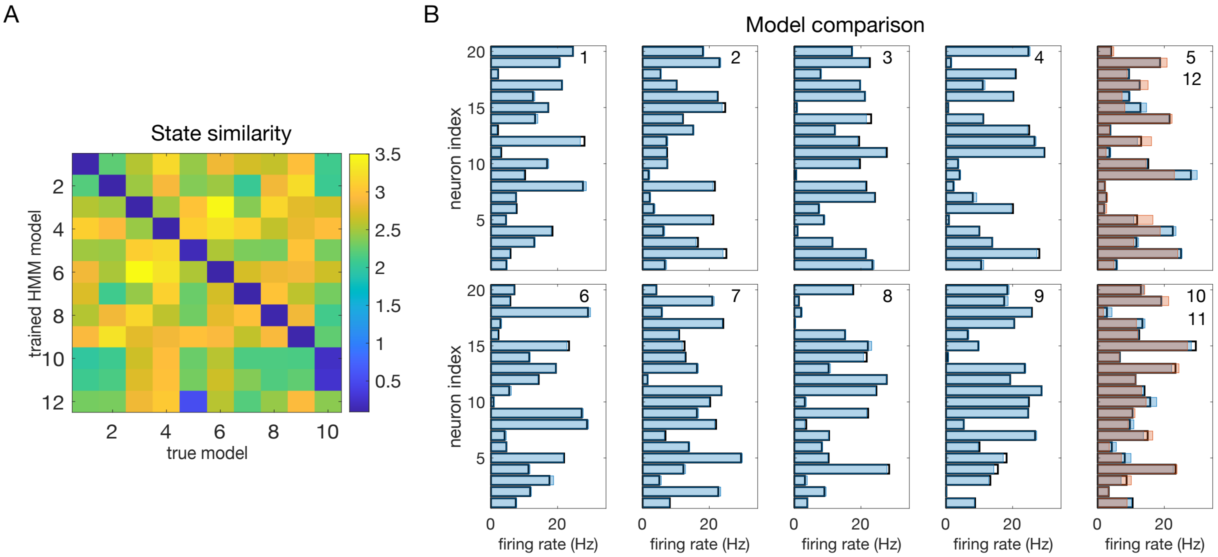

Figure 4: States comparison between trained HMM and true model (same models and dataset used in Fig. 2). **A.** Similarity between the true states and the states of the HMM model trained with 12 states. The entry in row i and column j is the Euclidean distance between the firing rate vector of state i of the trained model and the firing rate model of state j of the true model, with the states' labels in the two models matched for maximum similarity (see the text). **B**. Each panel shows the firing rate vectors of the true model (black rectangles) and the firing rate vectors of the matching 10 states in the trained HMM model (blue filled rectangles). For states 5 and 10, the firing rate vectors of states 11 and 12 of the model trained with $m = 12$ states are also shown (red filled rectangles).

We next asked the question as to why the model with 11 and 12 states seem to do as well as the model with $m = 10$. One guesses that the reason is the over-segmentation described in the manuscript, i.e., the extra states may 'duplicate' some of the original 10 states. To confirm this hypothesis, we compared the Euclidean distances between the firing rate vectors of the true model and the model trained with $m = 12$ states. We first matched the states of two different models according to their similarity: state i of model A was matched to state j of model B if the Euclidean distance $d(i, j)$ between the corresponding firing rate vectors was lowest across all distances $d(a, b)$ with $a \in A, b \in B$. This was accomplished using the so-called Hungarian algorithm (Kuhn, 1955) as implemented by Matlab's function `matchpairs`.

After running this procedure, the states were relabeled so that the first 10 states in the two models are most similar to each other while different states are less similar. The similarity matrix is shown in Fig. 4A. The high similarity (blue diagonal) of the first 10 states confirms that the states were correctly matched. As for the extra states, 11 and 12, of the trained HMM model, they were very similar to states 10 and 5 of the true model, respectively.

This is confirmed in Fig. 4B, which shows the comparison, neuron by neuron, between the states of the two models. Shown are the firing rate vectors of the true model (black rectangles), the firing rate vectors of the matching 10 states in the trained HMM model (blue filled rectangles), and the firing rate vectors of states 11 and 12 of the trained model (red filled rectangles) superimposed, respectively, onto states 10 and 5 (which are most similar to them). The true states are estimated extremely well by the trained HMM model, except for states 5 and 10, that are estimated, less well, using 2 states for each. This is because the trained HMM, being forced to contain 2 extra states, i) finds versions of the same state that

are most dissimilar across different occurrences (e.g., states 5 and 12), ii) splits the observations of these states into 2 sets (those best matched by state 5 vs. those best matched by state 12), and iii) assigns the corresponding states to the two sets. For example, neuron 9's observed firing rate must typically exceed the true firing rate when assigned to state 5, while it underestimates it when assigned to state 12. Clearly, this process results is similar model performance, i.e., in similar ρ and LL_v values, when the model is trained with $m = 10$ vs. $m = 12$ states, as shown in Fig. 2 and Fig. 3.

The contents of this reply (including the figures) has been accommodated in a new section of the manuscript, Sec. 2.7, "Full model comparison".

3) Similarly, when looking at the number of initial conditions, in such a situation the models being compared have the same number of states so LL is a good measure of the accuracy of the model. It would be nice to see a histogram of LLs achieved from different initial conditions, or to see the typical number of times the same optimal state is reached with different numbers of initial conditions. That is, when the authors say they see little improvement moving to 1000 i.c.s, is that because the best model found with 100 i.c.s is now reached 10 or more times, or because different models with similar LL are reached? Just focusing on how often the "correct no. of states in the model" is found leaves out plenty of info. That is, models that reach the same no. of states can find very different sets of states and probabilities, so how accurate are those sets of states, and how often are they the same across i.c.s?

To answer this question, we performed the same analysis of the previous query on the dataset used for Fig. 9 of the original manuscript (now appearing as Fig. 11), using the best model trained with 1,000 initial conditions as the true model. A ρ value close to 1 means that, if we converge to the same number of hidden states using 100 initial conditions rather than $1,000$ initial conditions, then the same states, state transitions and transition times are inferred. We show in Fig. 5A that this is indeed the case.

For comparison, randomly shuffling the states' firing rates and the off-diagonal transition probabilities gives a higher value of $\rho \approx 1.2 \pm 0.1$ which, as we know from Figs. 2-3, gives a poor match to the true model. The probability of getting the trained ρ values under the shuffled model is less than 0.009. Fig. 5B shows that the same analysis for the model trained with $1,000$ initial conditions (compared to the 'true' best model with $1,000$ initial conditions); the statistics are similar to Fig. 5A, showing that any model trained with $1,000$ initial conditions with the same number of states will match the best model.

We have reported these results in Sec. 2.8 of the revised manuscript ("The role of the initial parameter guesses") and in Supplementary Figure 2.

4) P. 16, "a subset of states were ... difficult to separate": this can be quantified. Each time the state is visited there is a measured firing rate (number of spikes divided by duration) that is different from the fiducial/veridical firing rate for each cell. One could look at the distributions of these data points and see if the clusters overlap for different states and mention their separability (this has been used to determine optimal number of states in some prior works – they stop adding states when there is overlap in distributions).

To measure the overlap between states we use the d' of detection theory:

$$d' = |\mu_1 - \mu_2|/\sigma, \qquad (3)$$

where μ_i is the mean in group i, $\sigma = \sqrt{((n_1 - 1)\sigma_1^2 + (n_2 - 1)\sigma_2^2)/(n_1 + n_2 - 2)}$ is the pooled standard deviation, and n_i is the number of samples in group i. As a rule of thumb, $d' \approx 1$ tells that the difference

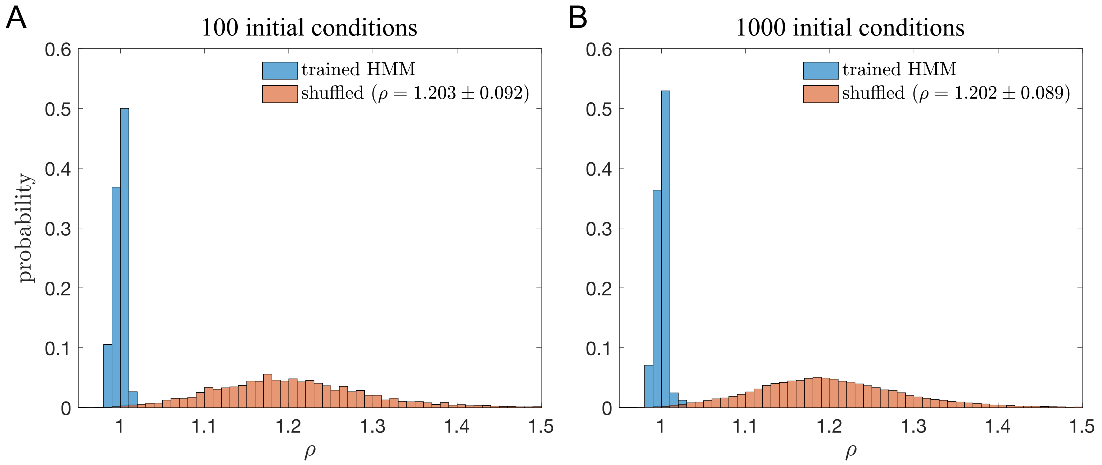

Figure 5: $\rho = D(\Theta)/D(\Theta^*)$ for the models in Figure 11 of the main manuscript, where Θ^* is the best model trained with $1,000$ initial conditions (*in lieu* of the true model). **A**. Histogram of the ρ values when Θ is the model trained with 100 initial conditions (blue) compared with the histogram of the same model after shuffling its states' firing rates and off-diagonal transition probabilities (same as Fig. 2A in this letter; 100 shuffles for each trained model). The ρ values were narrowly distributed around 1, with a probability of 0.0082 of getting the largest ρ value (or a smaller value) under the shuffled model. **B**. Same as panel A with Θ being the model trained with $1,000$ initial conditions. The probability of getting the trained ρ values under the shuffled model is 0.0098.

between the distributions is of the same order as their (pooled) variability, and therefore the samples may come from the same distribution. Then, arguably, it may be difficult to separate the states.

We show in Fig. 6 the box plots of d' values for each pair of states of the model of Fig. 4 trained with 12 states. Each bin of data was assigned to the state with the largest posterior probability, and the actual firing rates were measured in each state occurrence. This gave a distribution of firing rates for each neuron in a given state, and d' for each pair of states was computed for that neuron. Finally, for each pair of states, the distributions of d' values across neurons were plotted as box plots in Fig. 6. The box plots show meaningfully smaller values of d' (indicating larger overlap) only for the state pair $(5, 12)$ and the state pair $(10, 11)$ (red box plots), confirming the results of Fig. 4. According to this analysis, these state pairs may not be 'separable' by an HMM with 12 states trained on surrogate data generated by this model.

However, similarity of states and their separability by HMM are not always clearly linked as in the previous example. When we repeated the analysis on the MMPP datasets of Fig. 8 of our manuscript, we found no obvious similarity among the seven states of the model used to generate the data (Fig. 7B below). We chose $m = 7$ since this was the optimal number of states according to BIC (Fig. 8A of manuscript); Fig. 7A below confirms, based on the ρ index of similarity, that the most parsimonious model matching the true model's performance has 7 states.

Based on this, we decided that a link between similarity and separability is not always reliable. In particular, we no longer feel confident about the algorithm we had proposed as a procedure to reduce overfitting of experimental datasets, and have removed the corresponding section in the manuscript. In its place, we comment on the reason why BIC fails to capture the ground truth as the number of states grows larger:

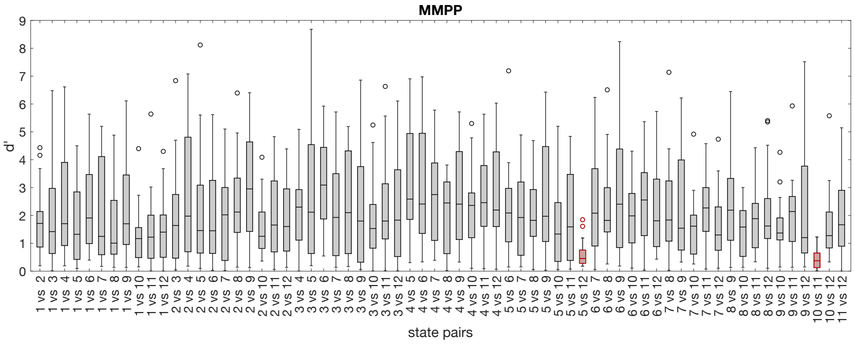

Figure 6: Box plots of d' values between the distributions (across neurons and states occurrences) of the firing rates of pairs of states in each neuron, for the model with $m = 12$ of Fig. 4. Only the state pairs $(5, 12)$ and $(10, 11)$ (shown in red) have narrow d' distributions with small median, confirming the similarity results of Fig. 4.

"*BIC eventually underestimates the true number of states because additional* $\ln D$ *terms in Eq. 1 subdue the increase in LL, resulting in a higher BIC than achieved with a smaller number of states. (...) The opposite occurs for AIC, where adding states causes a smaller additional penalty compared to the gain in LL.*"

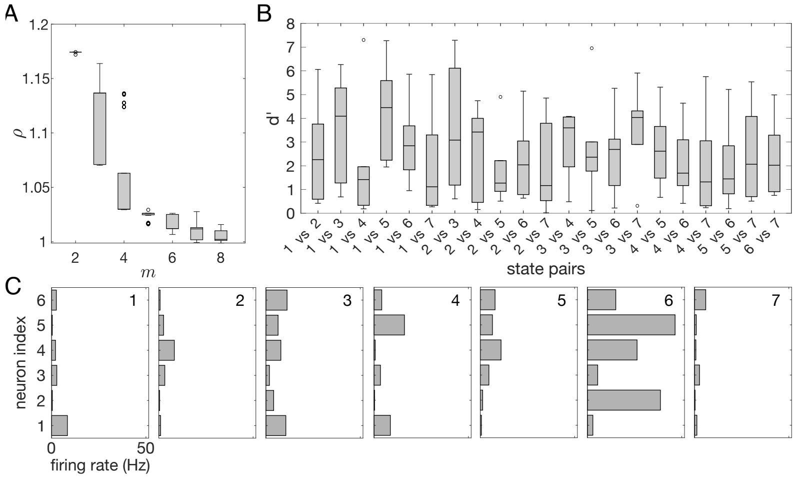

Figure 7: **A.** $\rho(m) = \rho(\Theta(m), \Theta_{true})$ between the true model and an sPHMM with m states trained on an MMPP generated by the true model (same keys as Fig. 2A). The true model was an sPHMM with 7 states, the best model (according to BIC) trained on the EXP dataset of Fig. 8A of our manuscript. ρ decreases with m until m reaches the true number of states $m^* = 7$, with similar $\rho \approx 1$ values for $m = 7$ and 8. **B.** Box plots of d' values between the distributions of firing rates of pairs of states for the true model of panel A (same analysis as Fig. 6). **C.** Hidden states of the true model of panels A and B.

Minor comments:

First line of intro: "random transitions" – "randomly timed" is better as the structure of the HMM shows that they are constrained to where they can go, sometimes in a reliable sequence. (The word "random" without a qualifier suggests equally likely to go to any state).

Done, thank you for this observation.

Fig 2 caption "on representative of all three" has a grammar error.

Thank you. We have replaced "on representative of all three datasets" with "on three datasets".

p.7 bottom. "But how to . . . strategy?" is not a sentence

Thank you. We have replaced this sentence with "But how can one extract the best value of m according to this strategy?"

p. 8 "suggest to use" is incorrect grammar

We have replaced "suggest to use" with "suggest using".

Reviewer 2

From what I understand, the paper basically implements a minimum threshold on self-transition elements of the transition probability matrix to solve the problem of overfitting when applying HMMs to neural data. With my background in applying HMMs to fluorescence microscopy data, I was able to mostly understand the paper however the presentation can improve a bit to make it more pedagogical. At the moment, I suspect more biology oriented audience would have a hard time understanding everything in the paper. I have following additional concerns.

We thank the reviewer for this feedback. To improve the readability of the manuscript, we have given informal descriptions of the methods as soon as they are introduced, including the formulae for the information criteria and a discussion of the physical meaning of the threshold for the sticky algorithm. We have also anticipated to the Introduction the notions of hidden states, decoding, and rapid state switching, aided by 4 new panels in Fig. 1. This figure now serves many purposes: it shows examples of segmentation of spike data by HMM, examples of hidden states and state decoding for two decoding methods (Viterbi and posterior decoding), it illustrates the problem of rapid state switching and shows that it does not depend on the decoding method. By doing this, we hope we have provided a more readable account of our methods and results.

1. In Fig 1. and others, it is visually hard to understand the color coding of states. The caption is not clear about what the colors represent in the context of HMM. I am guessing they represent one of the possible states of the system. In the caption, they should be connected to the m-dimensional state space mentioned in the text.

We have added 2 bottom panels to Fig. 1 to show the hidden states as vectors of firing rates across neurons, in the same color code as they appear in the raster plots of the top and middle panels. We hope to have now clearer descriptions of these notions, both in the caption and in the Introduction, where these notions are now first discussed.

2. Page 3, Intro section, second paragraph: I am confused about the number of hidden states being called the order of HMM. Usually, in the physics literature I have read, order refers to how many hidden states in the time sequence of hidden states the observations depend on. For instance, in the simplest single-order HMM, an observation w_n at time t_n would depend on the state s_n only, that is, a memory-less process. In a second-order HMM, w_n may depend on two states like s_{n-1} and s_n. Not a memory less process. Am I missing something?

The reviewer is correct that order of a Markov model (and of an HMM) can refer to the number of states upon which the current transition depends. Unfortunately, the term "order of an HMM" can also mean the number of hidden states, especially in the context of inference (where "order selection" refers to inferring the number of hidden states). Examples are Mackay (2002); Stoica and Selen (2004); Cappe et al. (2005); Zheng et al. (2019); Lin and Song (2022). To avoid confusion, we have removed the term 'order' when used with this meaning, and have replaced it with "the number of hidden states".

3. The authors come up with the value of 0.8 for the minimum threshold. The physical reasoning for this choice should be mentioned the first time this value it appears and not until much later in the Appendix A.

We now state that a 0.8 threshold corresponds to mean state durations of at least 250 ms the first time this threshold is mentioned (Section 2.2, "Algorithms"), and provide a full discussion in Section 2.4, "Temporal state persistence".

4. I am also concerned about the mathematical innovation in this paper. Most of the techniques used in the paper, AIC, BIC, sticky priors, et cetera are well established in the literature even in biophysics community. This means the paper is mostly about the application of these techniques to neural dynamics. So, the paper would meet the criteria for publication as long as the application is novel/important enough, which hopefully other reviews will comment on.

We believe that our paper meets the usual criteria for publication in a reputable Journal. Although sticky priors have been discussed in the literature, they have never been used as in our algorithm. Sticky priors are usually meant in the sense of our Dirichlet prior, i.e., as soft-bounds that do not guarantee the absence of rapid state switching (as we show in our paper). Moreover, we are not aware of systematic discussions of the problem of using cross-validation or information criteria when selecting an HMM. Although cross-validation fails and information criteria are not theoretically justified for HMMs, researchers keep using these methods without knowing or discussing what potential error they might commit. To make things worse, there is confusion about which selection method is best; some authors use cross-validation, some use BIC, others AIC and others still use other criteria. When using cross-validation, some authors select the number of states by visual inspection of the loglikelihood curve's elbow, while others guess the beginning of the plateau. As we show in our paper, these methods cannot be trusted. Another important topic that we never see discussed in the literature is the dependence of model selection on the number of random initial guesses, with most groups (ours included) often choosing as little as 5 to 10 random initial conditions. Our work was motivated by frustration with this whole state of affairs. To our knowledge, we offer the first systematic study of all these problems and give clear guidelines on how to select an HMM. Moreover, we present the first algorithm that *guarantees* the absence of rapid state switching, if training converges to a solution; and if it doesn't, this is a strong indication that the data are not to be described by an HMM. Although customized to spike data, our results and methods can be used for virtually any temporal series analyzed with HMM, and therefore it has a wide

scope and can benefit scientists working in many fields. Finally, we believe that although it is important to introduce new methods in the literature, it is just as important to understand how to correctly apply existing methodology that is widely used and can potentially lead to malpractice in data analysis.

5. It would be nice if the authors can comment on other nonparametric HMM methods such as infinite Hidden Markov Models (iHMM) with unknown number of states for the application in question. They do some of this in the discussion but additional comments would be helpful.

We have added a full paragraph in the Discussion (Section 3.5, "Alternative approaches") about the infinite-HMM and the comparison with our sPHMM.

References

Beal M, Ghahramani Z, Rasmussen C (2002) The infinite hidden markov model In Dietterich T, Becker S, Ghahramani Z, editors, *Advances in Neural Information Processing Systems*, Vol. 14, pp. 577–85. MIT Press.

Cappe O, Moulines E, Ryden T (2005) *Inference in hidden Markov models.* Springer series in statistics. New York, NY: Springer.

Kuhn HW (1955) The hungarian method for the assignment problem. *Naval Research Logistics Quarterly* 2: 83–97.

Lin Y, Song X (2022) Order selection for regression-based hidden markov model. *Journal of Multivariate Analysis* 192: 105061.

Mackay RJ (2002) Estimating the order of a hidden Markov model. *Canadian Journal of Statistics* 30: 573–589.

Stoica P, Selen Y (2004) Model-order selection: a review of information criterion rules. *IEEE Signal Processing Magazine* 21: 36–47.

Zheng J, Huang J, Tong C (2019) The order estimation for hidden markov models. *Physica A: Statistical Mechanics and its Applications* 527: 121462.
